# Insulin Receptor Signaling in the GnRH Neuron Plays a Role in the Abnormal GnRH Pulsatility of Obese Female Mice

**DOI:** 10.1371/journal.pone.0119995

**Published:** 2015-03-17

**Authors:** Sara A. DiVall, Danny Herrera, Bonnie Sklar, Sheng Wu, Fredric Wondisford, Sally Radovick, Andrew Wolfe

**Affiliations:** Department of Pediatrics, Johns Hopkins University, Baltimore, Maryland, United States of America; University of Córdoba, SPAIN

## Abstract

Infertility associated with obesity is characterized by abnormal hormone release from reproductive tissues in the hypothalamus, pituitary, and ovary. These tissues maintain insulin sensitivity upon peripheral insulin resistance. Insulin receptor signaling may play a role in the dysregulation of gonadotropin-releasing hormone (GnRH) secretion in obesity, but the interdependence of hormone secretion in the reproductive axis and the multi-hormone and tissue dysfunction in obesity hinders investigations of putative contributing factors to the disrupted GnRH secretion. To determine the role of GnRH insulin receptor signaling in the dysregulation of GnRH secretion in obesity, we created murine models of diet-induced obesity (DIO) with and without intact insulin signaling in the GnRH neuron. Obese control female mice were infertile with higher luteinizing hormone levels and higher GnRH pulse amplitude and total pulsatile secretion compared to lean control mice. In contrast, DIO mice with a GnRH specific knockout of insulin receptor had improved fertility, luteinizing hormone levels approaching lean mice, and GnRH pulse amplitude and total secretion similar to lean mice. Pituitary responsiveness was similar between genotypes. These results suggest that in the obese state, insulin receptor signaling in GnRH neurons increases GnRH pulsatile secretion and consequent LH secretion, contributing to reproductive dysfunction.

## Introduction

Obesity and conditions with hyperinsulinemia such as type 2 diabetes mellitus, metabolic syndrome, and polycystic ovary syndrome (PCOS) are often accompanied by infertility in females [1]. The role of hyperinsulinism in female reproductive dysfunction is undisputed [2]. Insulin is a co-gonadotropin with LH, causing increased steroidogenesis and altered follicular maturation in animal models [3] and women with PCOS [2]. The effect of hyperinsulinemia on central reproductive tissues such as the pituitary and hypothalamus is not well defined.

Experiments using various animal models and experimental paradigms have suggested a role of insulin signaling in central reproductive function. Central infusion of insulin to diabetic male sheep [4] or female rats [5] is associated with increased LH secretion; a similar study in male rats did not show this effect of insulin [6]. This suggests a sexual dimorphism in the response of the central reproductive axis to perturbation of insulin in rodents. Female but not male mice with absence of the insulin receptor in the brain exhibit hyperinsulinemia and peripheral insulin resistance with lower LH levels and subfertility, indicating a neuronal action of insulin to regulate LH release in female rodents [7]. Deletion of leptin and insulin receptors in POMC neurons cause hyperinsulinemia and insulin resistance with higher LH and testosterone levels in intact female mice compared to control [8]. Although it is possible that disrupted POMC neuron function contributed to the altered reproductive hormone levels, the hyperinsulinemic milieu generated may have also contributed to the observed phenotype.

Candidate neurons that may be directly involved in central reproductive function in an altered insulin environment include the GnRH neuron and the kisspeptin neuron. Tissue specific knockout studies indicate that absence of the insulin receptor in GnRH neurons [9] or kisspeptin neurons [10] does not alter LH levels in adult lean mice. The function of these altered neurons in the chronically hyperinsulinemic state, however, was not tested. We previously reported that hyperinsulinemia in diet-induced obese mice is associated with LH hypersecretion, female infertility, and hypertestosteronemia [3, 11]. Disruption of the insulin receptor (IR) specifically in gonadotrophs partially restored fertility and LH levels [11], while disruption of the IR in ovarian theca cells partially restored fertility and decreased androgen levels; LH levels were unaffected [3]. Hypothalamic dysfunction may partially account for the observed phenotype in obese female mice, as deletion of IR signaling in the gonadotroph did not completely restore reproductive function. Furthermore, the decreased testosterone levels associated with the lack of IR signaling in the theca cell was not associated with a decrease in LH levels, suggesting that other factors may be contributing to LH hypersecretion (3). To determine the role of IR in the reproductive neuroendocrine dysfunction associated with obesity in female mice, we used a mouse model of IR deletion in GnRH neurons [9] and induced hyperinsulinemia with diet induced obesity.

## Methods

### Animals

Generation and tissue specificity of the GnIRKO mice is as described previously [9]. The GnRH mice developed by this laboratory [12] were bred onto an outbred strain resulting in a mixed CD1, B129, and C57Bl/6J background. The IR floxed mice were a gift of CR Kahn [13] and were also bred onto a similar mixed background. Diet induced obese (DIO) mice were generated as previously described [11] in which 2 month old female mice were fed 60% high fat diet (HFD; D12492 Research Diets, New Brunswick, NJ) for 12 weeks, and then the metabolic and reproductive phenotypes of the mice were assessed. The HFD was maintained throughout phenotype assessment. DNA extraction and primers for genotyping are described previously [9]. All experimental animals were from 2 founder matings. Mice with deletion of the IR in GnRH neurons (GnRHCre+/-; IR fl/fl) are referred to as GnIRKO in this manuscript. Littermates with genotype of GnRHCre-/-; IR fl/fl and mice with genotype of GnRHCre+/-; IR fl/wt were used as controls. Isoflurane was used for sedation or euthanasia prior to survival procedures or terminal procedures. All procedures were performed with approval of the Johns Hopkins Animal Use and Care Committee (protocol #MO11M255).

### Fertility assessment

Vaginal cytology was performed and estrous cycling was analyzed for 13 days, using the method of Nelson et al [14]. Proestrus was assigned when nucleated cells predominated; estrus was assigned when cornified cells predominated; metestrus when cornified cells and leukocytes were present; and diestrus when leukocytes predominated. To assess fertility, 5 month old female mice were entered into a 7 day breeding rotation individually with proven fertile male mice, and fertility rate was presented as a percentage of the four mating trials that resulted in pregnancy as described in [11]. The presence or absence of vaginal plugs was assessed daily after male introduction.

### Histology

The ovary was dissected from mice in diestrus and fixed in 10% formalin phosphate buffer and sectioned to 5 μm thickness in its entirety by the Histology department of the Johns Hopkins Medical Laboratories. Every 10th section was collected, and ovarian sections were stained with hematoxylin and eosin. The corpora lutea were counted and examined with a Zeiss microscope. Sections were photographed with an AxioCamMR camera and exported to AxioVision software.

### Insulin or GnRH Stimulation

Baseline blood was collected via mandibular bleed prior to intraperitoneal injection of 1.5 units/kg of regular insulin (Lilly) or subcutaneous injection of 100 ng/kg of GnRH (Sigma). Blood was collected 40 minutes after insulin injection or 10 minutes after GnRH injection as described in [11].

### Hypothalamic explants

Age-matched mice were sacrificed at metestrus or diestrus and the hypothalamus harvested and dissected using the method of Woller et al [15]. Briefly, the mediobasal hypothalamus was delimited laterally by the hypothalamic fissures, anteriorly by a cut anterior to the optic chiasm, and posteriorly by the rostral portion of the mammillary bodies [15]. The hypothalami were then placed in 500 μL culture with phenol free DMEM containing 25 mM glucose and supplemented with 0.1% BSA, 0.006% bacitracin, and glutamine. Every 7 minutes, media was collected and replaced according to the method originated by Bourguignon in rats [16] and later performed in transgenic mice with an incubation period of 6 hours [17]. Potassium chloride (60mM) to induce neuropeptide release was added to the media in the last 14 minutes of the experiment. Samples were immersed in 95 degrees for 5 minutes, then snap frozen and stored at-70 degrees until RIA.

### Hormone Determination, quantitative real-time PCR, and Radioimmunoassay

LH and FSH were measured by Luminex assay (Kit RPT-86K; EMD Millipore, Germany) as previously described using 10μL of serum [9]. Insulin and leptin from 6 hour fasted mice were measured with Luminex assay using magnetic bead kit MADKMAG-71K according to the manufacturer’s instructions using 10 μL of serum (EMD Millipore, Germany). Testosterone was measured via radioimmunoassay by the University of Virginia Center for Research in Reproduction, Ligand Assay and Analysis Core using 60 μL of serum. Glucose was measured by UltraTouch meter (Lifescan). GnRH gene expression was determined using quantitative real-time PCR as described previously [18, 19]. GnRH radioimmunoassay was performed using rabbit anti-GnRH (gift of T. Nett), 4,000 cpm of ^125^IGnRH (University of Wisconsin Primate Research laboratory), and goat anti-rabbit secondary antibody (Bachem). Synthetic GnRH used for standards and reference samples was purchased from Bachem. Sensitivity of the assay was 0.2 pg/sample at 95% binding (= 1.0 pg/mL). The antibodies and ^125^IGnRH were previously tested and validated in rodent hypothalamic explants and no cross reactivity was noted [15]. Intra- and Interassay coefficients of variations were 6% and 12%, respectively.

### Pulse Detection and statistical analyses

To identify GnRH pulses, the Cluster algorithm [20] was used. Pulse peaks were determined as two points per peak flanked by two nadir points and a 5.0% significance cutoff level as determined by up-stroke and down-stroke t-statistic value of 2.0. The first 30 minutes of incubation (equilibrium phase) and last 14 minutes of incubation (KCl administration) were excluded from the final analysis. After Cluster analysis, one-way ANOVA was used to determine significant differences in pulse frequency, amplitude, and cumulative area.

Data passed tests for normality and were analyzed using one-way ANOVA and Tukey’s post-hoc test using GraphPad4 software (GraphPad, Inc., San Diego, CA). Weight data, estrous cycle analysis, and glucose tolerance testing were analyzed using two-way ANOVA with Bonferroni post-hoc tests. Corpora lutea numbers were compared using student’s t-test after testing for normality.

## Results

### DIO female mice exhibit weight gain and metabolic perturbation

**Fig 1:**
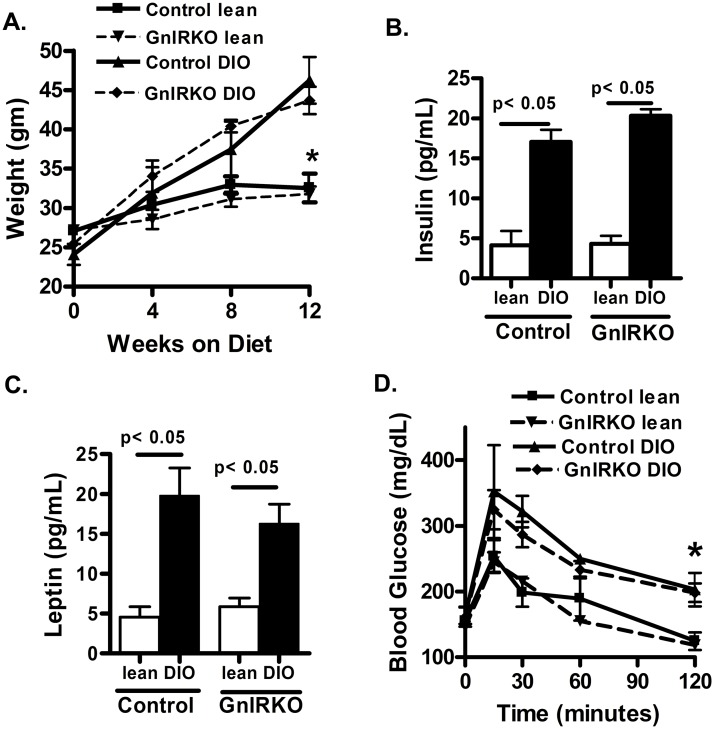
Metabolic function is similar between control and GnIRKO mice. (A) Body weight in lean mice maintained on regular chow and DIO mice introduced high fat diet at 8 weeks of life. (B) Serum insulin and (C) serum leptin after a 6 hour fast in mice fed high fat diet for 12 weeks. (D) Glucose tolerance test. Values are mean ± SEM. n = 6–8 each group *, p <0.05.

To determine the role of obesity on IR signaling in the GnRH neuron, female mice were administered 60% fat diet starting at 8 weeks of life. GnIRKO DIO mice exhibit similar weight gain (Fig. 1A) and metabolic perturbation compared to control DIO mice (Fig. 1B-D). Fasting plasma insulin (Fig. 1B) and leptin (Fig. 1C) were determined in mice at approximately 5 months of age, when the DIO group had been administered the high fat diet for 12 weeks. As expected, lean mice had lower serum insulin than DIO mice. There was no significant difference in serum insulin between genotypes in the lean or DIO state. An intraperitoneal glucose tolerance test indicated that the DIO mice of each genotype were equally glucose intolerant compared to their lean counterparts (Fig. 1D). Collectively, these results indicate that absence of the insulin receptor in GnRH neurons does not affect peripheral glucose metabolism and that the degree of hyperinsulinemia was similar between genotypes.

### Fertility is improved in GnIRKO DIO mice

**Fig 2:**
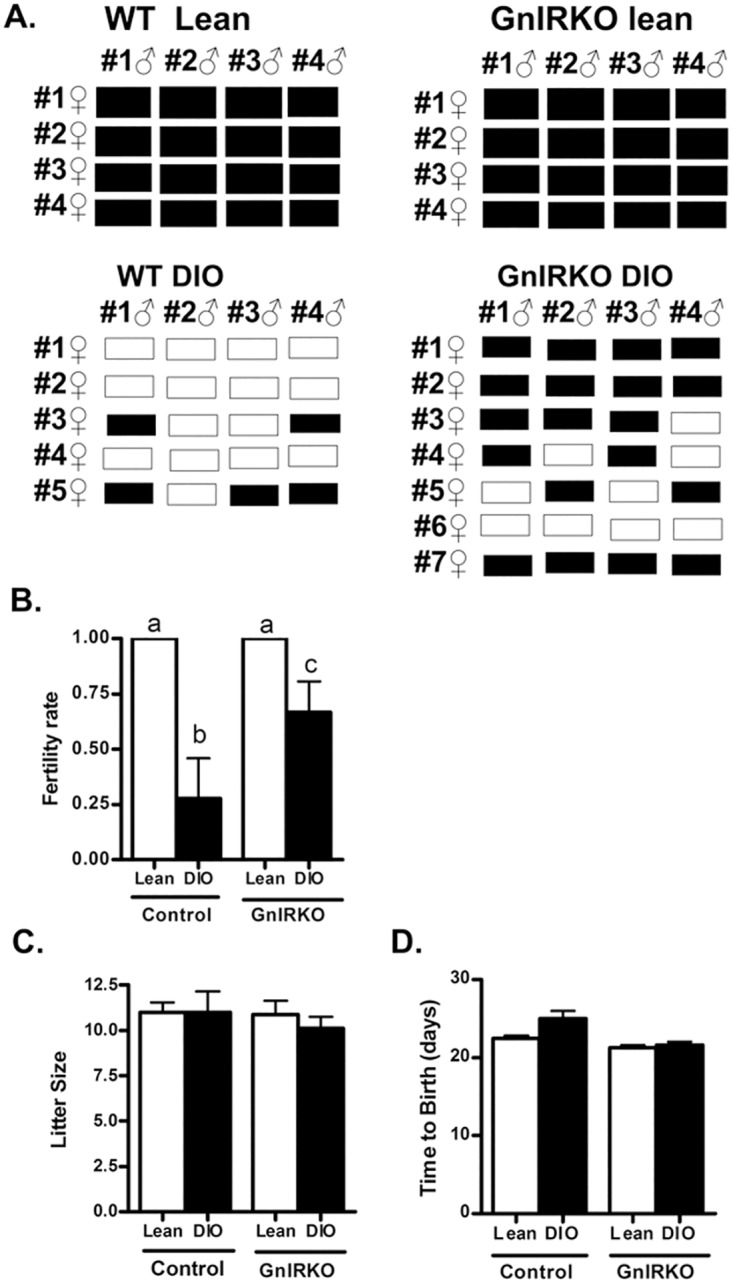
Impaired fertility of obese female mice is improved by deletion of IR in GnRH neurons. (A) Matrix of the breeding study. Each row represents a female, with each bar representing an individual pairing with the male of the column. A black bar indicates a resultant pregnancy, whereas a white bar indicates no pregnancy. (B) Calculated mating success rate under lean and DIO conditions. There is no statistical difference between groups with the same letters. (C) Resultant litter size and (D) Time to birth with observed pregnancies. Data are mean ± SEM.

Lean GnIRKO mice have normal fertility and estrous cycling [9], and wild-type DIO mice have impaired fertility that is improved upon deletion of the IR in the gonadotroph [11] or theca cell [3]. To assess whether IR signaling plays a role in neuroendocrine reproductive dysfunction of obese mice, we assessed fertility in GnIRKO DIO mice. To do this, between four and seven female mice of each group were paired individually with four different males of proven fertility. The males alternated between pairings with DIO and chow-fed females in order to minimize any effects of diet on male reproductive function. Vaginal plugs were observed upon pairing. Lean female mice of either genotype consistently produced litters, while control DIO mice had an impaired ability to produce pups, as shown in the mating matrix (Fig. 2A). Lean mice of either genotype had a fertility rate of 1.0, while control DIO mice had an average fertility rate of 0.25 and the GnIRKO DIO mice had an average fertility rate of 0.67 (Fig. 2B). The fertility rate of the control DIO mice is similar to that previously reported by this laboratory [3, 11]. Pregnancies in the GnIRKO DIO mice had a similar time to birth after introduction of the male and similar number of pups (Fig. 2C and 2D).

**Fig 3:**
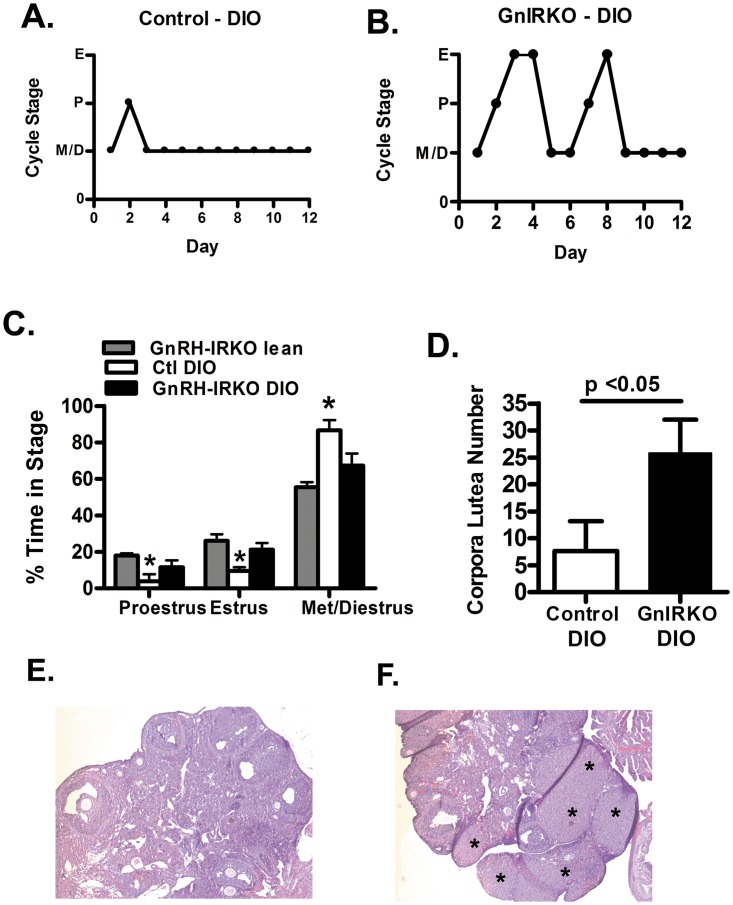
Impaired ovulation is improved by deletion of IR in GnRH neurons. (A). Graphic representation of the estrous cycle in control DIO (left panel) and GnIRKO (right panel) determined by vaginal cytology for 13 days. (B) Time in each estrous cycle phase. (C) Total corpora lutea counts for control and GnIRKO DIO mice. Values are mean ± SEM. n = 4–6 each group. * p<0.05. (E) Representative control DIO ovary section. (F) GnIRKO ovary section. Asterisk denotes corpora luteum.

Lean GnIRKO mice have the same estrous cycle pattern as wild-type mice [9]. Upon assessment of the estrous cycle, control DIO mice generally exhibited persistent diestrus while GnIRKO DIO mice exhibited estrous cycling similar to lean mice (Fig. 3A and B). Assessment of the estrous cycle in 4 mice of each group is shown in Fig. 3C. The GnIRKO DIO mice had a similar estrous cycle pattern to the lean mice, spending an average of 67 ± 13 percent of the time in diestrus /metestrus, while control DIO spent a significantly greater percent of time in diestrus/metestrus. GnIRKO DIO mice had a greater number of corpora lutea than control DIO mice (Fig. 3D, E and F) indicating that these mice had more ovulatory events. That the control DIO mice had a low number of corpora lutea and thus ovulatory events is consistent with previous data [3, 11] and with the dominance of the diestrus state in their estrous cycling. The greater number of corpora lutea, thus ovulatory events in the GnIRKO group is consistent with the improved fertility and higher percentage of proestrus and estrus days upon estrous cycling analysis.

### Basal LH levels in GnIRKO DIO mice are intermediate between WT DIO and lean mice

**Fig 4:**
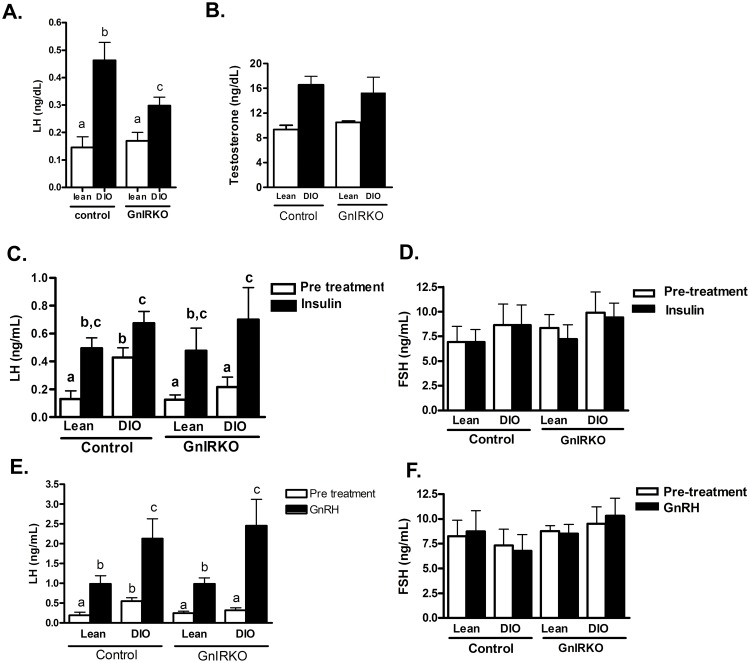
Basal LH is higher in control DIO animals. (A). Basal Luteinizing hormone (LH) levels across groups. (B) Basal testosterone. (C) LH and (D) FSH were determined at baseline and 40 minutes after injection of insulin. (E) LH and (F) FSH was determined at baseline and 10 minutes after injection of GnRH. Values are mean ±SEM. There is no statistical difference between groups with the same letters. n = 10–20 each group.

Basal LH levels were determined the morning of diestrus or metestrus. Basal LH levels were similar in lean mice of either genotype and significantly higher in control DIO mice (Fig. 4A) consistent with previous reports [3, 11]. The morning LH levels of GnIRKO mice, however, were intermediate between lean and DIO mice suggesting that insulin receptor in GnRH neurons plays a role in the elevated LH levels observed in the obese state. FSH levels were not significantly different between groups in the baseline or in the hormone stimulated state (Fig. 4D and F).

We previously reported that female mice with DIO have higher testosterone levels than lean mice [3, 11]. Testosterone levels were higher in control DIO mice (Fig. 4B), and GnIRKO DIO mice had similar testosterone levels to control DIO mice. This suggests that the decrease in basal LH in the GnIRKO mice was not due to differences in serum testosterone. Testosterone levels are not lower in the setting of lower LH, likely because of enhanced steroidogenesis due to insulin action on the ovary [2, 3].

Acute insulin administration increases LH release in female mice [11]. As shown in Fig. 4C, mice of both genotypes experienced an increase in LH release upon insulin administration indicating that acute insulin action on GnRH neurons does not significantly affect GnRH release to result in differential LH release. We previously demonstrated that insulin receptor in gonadotrope cells was necessary for the increase in serum LH upon acute insulin administration [11]; as GnIRKO mice have intact IR in pituitary gonadotropes, these findings are consistent with previous results. The stimulated LH level was not significantly different between lean and DIO animals. The higher baseline LH of the control DIO mice was again observed.

To evaluate gonadotrope function in the GnIRKO mice, a GnRH stimulation test was performed (Fig. 4D). After GnRH administration to lean mice, LH increased similarly in control and GnIRKO animals. In the DIO state, LH increased to higher levels in response to GnRH, as reported previously [3, 11]. The peak LH after GnRH was similar between control DIO and GnIRKO DIO animals, indicating that differential pituitary response does not account for the difference in basal LH observed between the control and GnIRKO DIO animals.

### GnRH secretion is enhanced in DIO mice

**Fig 5:**
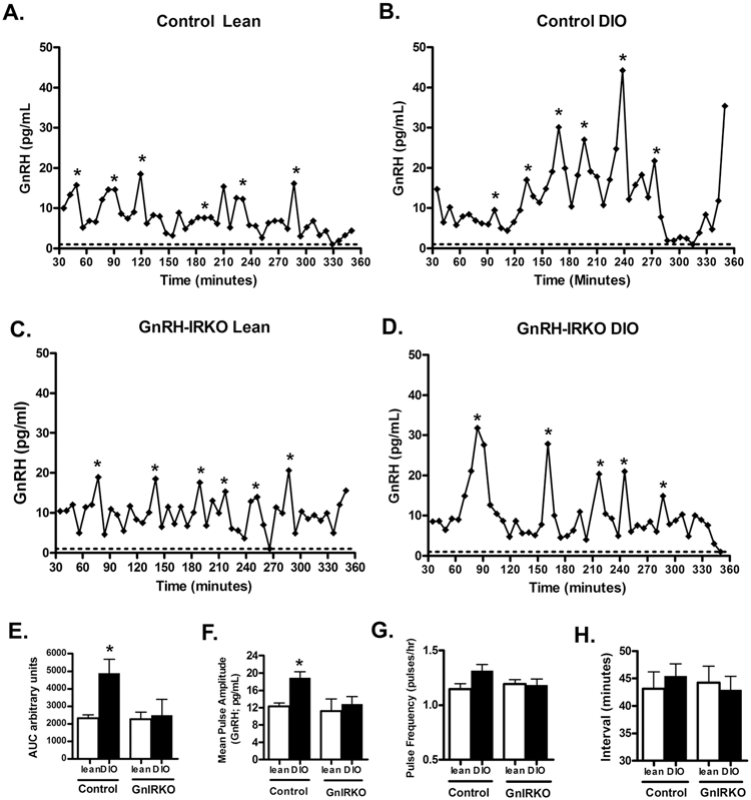
GnRH secretion is amplified in control DIO state. Hypothalami from mice were excised, incubated in *ex vivo* culture, and GnRH determined by RIA. Graphical representation of GnRH release from hypothalami of female (A) control lean (B) control DIO, (C) GnIRKO lean and (D) GnIRKO DIO. Asterisks denote significant peaks as determined by Cluster pulse analysis. Dotted line denotes the lower limit of the assay (1 pg/mL), (E) area under the curve (F) pulse amplitude, (G) pulse frequency, and (H) pulse interval for each group. Values are mean ± SEM. n = 7–8 mice per group. *, p< 0.05.

Given that basal LH levels of the GnIRKO DIO mice were intermediate between control DIO and lean mice and the stimulated levels were not different, we hypothesized that differences in GnRH secretion may contribute to the different baseline LH levels. To determine GnRH secretion patterns in mice of different genotypes and diet conditions, the hypothalami of mice were placed in *ex vivo* culture (15–17) and GnRH release from the hypothalami determined by RIA. Representative GnRH secretion patterns of the four different groups are shown; control lean (Fig. 5A), control DIO (Fig. 5B), GnIRKO lean (Fig. 5C) and GnIRKO DIO (Fig. 5D). Analysis of GnRH pulse pattern indicates that total GnRH secretion as represented by area under the curve, was highest in control DIO mice (Fig. 5E). In contrast, GnRH secretion of the GnIRKO DIO mice was significantly lower, approaching the total secretion of the lean animals. Mean pulse amplitude of the control DIO mice (Fig. 5F), was significantly higher than other groups while the pulse amplitude of the GnIRKO DIO mice was similar to the lean mice of both genotypes. Meanwhile, GnRH pulse frequency of the control DIO mice (Fig. 5G) trended higher, but was not significantly different from the pulse frequency of GnIRKO DIO mice. Thus, the higher total GnRH secretion of control DIO mice is predominantly due to higher pulse amplitude. Mean pulse interval was not significantly different between groups (Fig. 5H). GnRH gene expression is higher in control DIO mice than in GnIRKO DIO mice (data not shown), consistent with the hypersecretion of GnRH in the control DIO mice. It has been previously been shown that transgenic mice with the green fluorescent protein (GFP) in the GnRH neuron have altered GnRH pulsatility [21]. To test the function of the Cre gene on GnRH neuron function, we assessed the GnRH pulse pattern in GnRH Cre+/- IR fl/wt animals (S1 Fig.). GnRH pulse pattern, as determined by area under the curve, pulse amplitude, interval, and frequency were not different between Cre+ and Cre- in the lean or DIO state.

## Discussion

Obesity is marked by a complex metabolic phenotype that includes hyperinsulinemia and hyperleptinemia, with insulin and leptin resistance that each could contribute to the reproductive dysfunction associated with obesity. In this study, we provide evidence that obesity-associated hyperinsulinemia stimulates the GnRH neuron in female mice to enhance GnRH pulsatile secretion and subsequent LH secretion.

Absence of the insulin receptor on GnRH neurons does not affect reproductive function or hormone in the lean state (Fig. 2 and [9]) but is associated with a lesser degree of reproductive impairment in the DIO state (Figs. 2–5). Cumulatively, this data indicates that the hyperinsulinemic milieu of the DIO state directly activates the GnRH neuron to cause alterations in GnRH secretion and subsequent LH secretion. Insulin receptor signaling in the GnRH neuron does not entirely account for the disrupted reproductive dysfunction and hormonal alterations of the DIO state in females, as the LH levels and reproductive function of the DIO GnIRKO animals were intermediate between the WT DIO and lean states. Insulin receptor signaling in the pituitary gonadotroph contributes to the LH elevation and reproductive dysfunction of the DIO state in the female as indicated by the studies by Brothers et al [11]. Altered insulin signaling in other cells involved in the central regulation of reproduction may also play a role. Whether the altered LH associated with the obese state is entirely due to the IR signaling in the GnRH neuron and the gonadotroph can only be determined by the generation of a combined gonadotroph-GnRH neuron insulin receptor knock-out mouse. Generation of that mouse was beyond the scope of the present study.

The differences in GnRH secretion observed between lean, DIO control and DIO GnIRKO mice in the obese state (Fig. 5) imply that the GnRH neuron remains at least partially insulin sensitive in the hyperinsulinemic environment. The pituitary and ovary also exhibit insulin sensitivity in DIO; insulin-induced phosphorylation of Akt in these tissues is preserved in obese animals [22]. Given the homogeneity of the hypothalamus, we were unable to directly test the insulin sensitivity of the isolated GnRH neuron in the obese animal.

In the DIO state, LH is higher despite a higher testosterone level. This is in part due to impaired steroid negative feedback. Steroid negative feedback in the DIO state may be impaired due to insulin resistance in the proopiomelanocortin (POMC) neuron; lack of the insulin receptor in POMC neurons is associated with higher estradiol levels in females, and impaired ability of exogenous estradiol to lower gonadotropins in gonadectomized animals [23]. POMC neurons exhibit insulin resistance with obesity [24]. In our studies, DIO mice with deletion of IR in the GnRH neuron have GnRH secretion similar to lean animals despite similar androgen levels as DIO mice (Fig. 4). Thus, the hyperinsulinemic state impairs steroid negative feedback on GnRH thus LH secretion, but direct insulin action on the GnRH neuron also plays a role in the increased LH found in the DIO state.

The hormonal pattern that we observe in this model of obesity associated infertility does not completely recapitulate what is seen in humans. Higher LH levels compared to lean women are not observed in women with obesity and infertility, whether or not the infertility is associated with hyperandrogenemia [25, 26]. Women with obesity have a greater clearance of LH compared to lean women, which may account for these observations [27].

Insulin at low doses does not induce changes in GnRH secretion *in vitro* [28] while Burcelin et al noted that large doses of insulin increased GnRH secretion of neurons *in vitro* [29]. It is possible that hyperinsulinemia induces GnRH secretion via activation of the IGF-1 receptor which can bind insulin at high doses, but the reduction in GnRH secretion we observed upon deletion of the IR in the neuron suggests that activation is occurring via insulin receptor signaling. Additionally, the IGF-1 receptor is unlikely to be activated at the elevated insulin levels that are associated with obesity [30].

Insulin signaling may modulate GnRH peptide release either directly or indirectly. Insulin signaling can directly induce neuropeptide release at the distal neurite by increasing intracellular calcium concentration [31], even in the absence of neuronal firing. Alternatively, insulin signaling may crosstalk with G-protein signaling pathways to modulate calcium signaling and subsequent GnRH release. Insulin has been shown to potentiate G protein signaling by enhancing generation of inositol 1,4,5-trisphosphate and subsequent calcium flux [32]. Kisspeptin, the most potent stimulator of GnRH release, binds its G protein receptor to activate phospholipase C and inositol 1,4,5-trisphosphate pathways to affect calcium flux and membrane depolarization [33]. GnRH receptors, G-protein coupled receptors also present on GnRH neurons, primarily stimulate calcium flux via cyclic AMP activation, but also via inositol phosphate [34]. The effect of obesity on kisspeptin signaling is not clear. In some studies, obesity decreases kisspeptin expression upon DIO [35], but in other studies, overnutrition postnatally induces precocious kisspeptin expression [36].

The ovarian dysfunction and infertility observed in the DIO mouse model may be due to alterations in testosterone and LH in addition to insulin [3]. Transgenic mice with overexpression of LH have infertility and infrequent ovulation [37]. LH stimulates testosterone synthesis; treatment with high testosterone doses induces arrest of follicular maturation in mice [38].

In this study, DIO mice had greater GnRH secretion compared to lean and GnIRKO mice due primarily to higher pulse amplitude. While there was a trend towards greater pulse frequency, it was not statistically significant (Fig. 5). It is possible that shortening the time between sample collections in our experimental design may allow us to detect an increase in pulse frequency in the control DIO animals. However, the limitations of GnRH detection using the radioimmunoassay dictated the sample frequency in this study.

In summary, we provide evidence that the increased LH of obese female mice is partially due to insulin signaling at the GnRH neuron causing increased GnRH secretion. We previously reported that IR signaling in the pituitary gonadotroph [11] or ovarian theca cell [3] contributes to infertility and reproductive hormone dysfunction in female DIO mice. Together with the current findings, these studies indicate that hyperinsulinemia results in dysfunctional hormonal secretion at multiple levels of the reproductive axis.

## Supporting Information

S1 Fig Obese GnRH Cre+ fl/wt animals have similar GnRH secretion to obese Cre- animals. Hypothalami from mice were excised, incubated in *ex vivo* culture, and GnRH determined by RIA. (A) area under the curve (B) pulse amplitude, (C) pulse frequency, and (D) pulse interval for each group. Values are mean ± SEM. n = 4–6 mice per group.
(TIF)
