# Supplementary figures and images for: Insulin Receptor Signaling in the GnRH Neuron Plays a Role in the Abnormal GnRH Pulsatility of Obese Female Mice
Source: PLoS One. 2015 Mar 17;10(3):e0119995. doi: 10.1371/journal.pone.0119995 (PMC4363364; doi:10.1371/journal.pone.0119995)

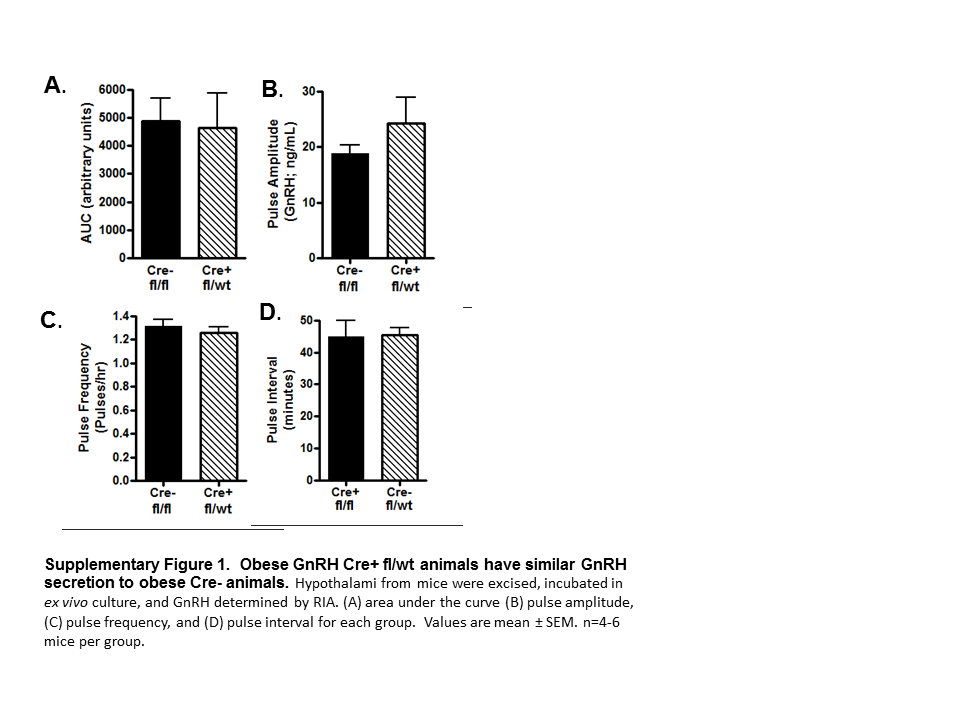

Supplement: S1 Fig — Hypothalami from mice were excised, incubated in ex vivo culture, and GnRH determined by RIA. (A) area under the curve (B) pulse amplitude, (C) pulse frequency, and (D) pulse interval for each group. Values are mean ± SEM. n = 4–6 mice per group. (TIF) [file pone.0119995.s001.tif]
